# Safety of disease-modifying therapies in multiple sclerosis: real-world data from the Austrian MS Treatment Registry (AMSTR)

**DOI:** 10.1007/s00415-025-13473-7

**Published:** 2025-11-19

**Authors:** Tobias Monschein, Helena Untersteiner, Markus Ponleitner, Nik Krajnc, Tobias Zrzavy, Gabriel Bsteh, Paulus Rommer, Barbara Kornek, Christian Enzinger, Franziska Di Pauli, Jörg Kraus, Michael Guger, Fritz Leutmezer, Thomas Berger, The Austrian MS Treatment Registry (AMSTR)

**Affiliations:** 1 Department of Neurology, Medical University of Vienna, Waehringer Guertel, 18-20, 1090 Vienna, Austria; 2 Comprehensive Center for Clinical Neurosciences and Mental Health, Medical University of Vienna, Vienna, Austria; 3 Department of Neurology, Medical University of Graz, Graz, Austria; 4 Department of Neurology, Medical University of Innsbruck, Innsbruck, Austria; 5 Department of Neurology, Medical Faculty, Heinrich Heine University Düsseldorf, Düsseldorf, Germany; 6 Department of Laboratory Medicine, Paracelsus Medical University and Salzburger Landeskliniken, Salzburg, Austria; 7 Department of Neurology, Pyhrn-Eisenwurzen Hospital Steyr, Steyr, Austria; 8 Medical Faculty, Johannes Kepler University Linz, Linz, Austria

**Keywords:** Multiple sclerosis, Disease-modifying therapy, Adverse events, Registry

## Abstract

**Background:**

In the therapeutic landscape of multiple sclerosis (MS), more than a dozen disease-modifying therapies (DMT) are currently available. In Austria, only certified MS centers are authorized to prescribe DMT and are obliged to enter patient data into the Austrian MS Therapy Registry (AMSTR). Here, we report the safety profiles of different DMT documented in this registry.

**Methods:**

Adverse events (AE) data from the AMSTR, collected at least biannually from August 2006 to July 2025, were extracted. AE were classified by system organ classes according to the Medical Dictionary for Regulatory Activities.

**Results:**

This analysis included 7913 patients (67.7% female, median age 37 years [IQ 29–45]), with the majority receiving dimethyl fumarate (2572/7913, 32.5%), followed by fingolimod (2129/7913, 26.9%) and natalizumab (2021/7913, 25.5%). For alemtuzumab, the most frequent AE were immune-related (41/90, 45.5%) including thyroid disorders (24/90, 26.7%). For cladribine, natalizumab, ocrelizumab and ofatumumab, infections constituted the most common AE (21/458, 4.6%; 123/2021, 6.1%; 27/698, 3.9%; 16/792, 2%). Gastrointestinal AE were most frequently observed with dimethyl fumarate and teriflunomide (364/2572, 14.2%, and 78/781, 10%, respectively), whereas AE related to the blood system were most common with sphingosine-1 phosphate receptor modulators (S1P-modulators; fingolimod: 245/2129, 11.5%). Regarding neoplasms, all DMT showed low rates, though S1P-modulators had the highest (fingolimod 31/2129, 1.4%—13/31 [42%] basal cell carcinomas).

**Conclusions:**

The safety data from the AMSTR do not reveal any new safety issues, particularly regarding neoplasms and infections. Hence, people with MS as well as their treating neurologists are reassured to continue treatment with DMT, as the benefit-risk profile of DMT could be reaffirmed.

**Supplementary Information:**

The online version contains supplementary material available at 10.1007/s00415-025-13473-7.

## Introduction

Multiple sclerosis (MS) is a chronic inflammatory disease of the central nervous system and affects over 2.8 million people worldwide with about 13,500 people in Austria [1]. While the therapeutic landscape of both relapse therapy and symptomatic therapy has not undergone major changes in the last decades, a wide range of disease-modifying therapies (DMT) has emerged over the last 30 years [2]. In general, DMT achieve their disease-modifying effect via immunomodulation and/or immunosuppression [3]. However, especially in the era of highly effective therapies and the related recommendation to the “hit hard and early” concept, the consideration of potential adverse events (AE) and risks is becoming increasingly important [4]. In addition to drug-specific AE, immunomodulation/suppression is especially suspected to increase the risk of infection and cancer [5, 6].

With the ongoing development of new and increasingly effective DMT, the challenges are not only growing for MS research and treatment centers, but they are also impacting healthcare systems on the whole [7]. Consequently, the Central European MS Expert Panel recommended the continuous use and evaluation of registries [8]. The Austrian Multiple Sclerosis Therapy Registry (AMSTR) was therefore set up in 2006, with over 100 MS centers accessing the registry and therefore covering the vast majority of people with MS (pwMS) in Austria [9].

Within the registry, data from approximately 8000 MS patients are being collected continuously and it therefore provides important information on safety, efficacy and treatment adherence in a real-world setting.

The aim of this study is to retrospectively analyze safety data on several DMT from the AMSTR in order to search for potential new safety signals in the therapy of MS on a real-world, nation-wide basis in Austria.

## Methods

In Austria, there is a comprehensive network of certified MS centres, hospital based as well as office-based ones. This network provides high-quality patient care and support throughout Austria and ensures appropriate application and monitoring of DMT treatments. The AMSTR was launched in 2006 to collect data on the efficacy and safety of approved DMT in a real-world setting and thereby monitor potential emerging long-term effects and risks [10]. In order to achieve the most comprehensive coverage of patients possible, data entry into the AMSTR is mandatory to fulfil the reimbursement requirements of the Austrian health insurance funds. Of note, the AMSTR is a secure web-based platform that requires online documentation during patient visits by treating neurologists in all Austrian MS centres. In order to improve the quality and reliability of the documented data, an independent data monitoring board was established [11].

The initial entry of baseline data into the AMSTR includes, besides others, MS onset and disease duration, relapses in the prior 12 months, expanded disability status scale (EDSS), and previous DMT use. The follow-up data include in particular EDSS, relapse rate and AE as well as change or discontinuation of treatment and is required to be captured every 3–6 months. In the case of treatment withdrawal, data regarding the reason for discontinuation are queried. In this study, data were obtained from the AMSTR in the period from August 2006 to July 2025.

AE`s in temporal and causal relation to drug therapy were classified by system organ classes according to the Medical Dictionary for Regulatory Activities (MedDRA). In this context, all events were categorized as severe AE (SAE) if life threatening or resulting in hospitalization, permanent disability, or death. Infusion-related reactions (IRR) were defined as any AE with onset during or  ≤ 24 h after the end of infusion.

### Ethics

The ethics committee of the Medical University of Vienna approved the study (ethical approval number: 1819/2021). As datasets were exported pseudonymously from the registry, the need for written informed consent from study participants for this work was waived by the ethics committee of the MUV.

### Data availability

De-identified data can be made available from the corresponding author upon reasonable request by a qualified researcher and upon approval by the data-clearing committee of the Medical University of Vienna.

### Statistics

Statistical analyses were carried out with IBM SPSS Statistics for Windows (Version 29 Armonk, IBM Corp., New York, NY, USA). Categorical variables are represented using both absolute and relative frequencies, while continuous variables are expressed as the median and the interquartile range (IQ).

## Results

The AMSTR included data of 7913 pwMS treated with DMT as by July 2025. The majority of this population was female (5359/7913, 67.7%) with a median age of 37 years (IQ 29–45) at AMSTR entry. The median baseline EDSS was 2 (1–3). Overall, 7541/7913 (95.4%) patients were presenting with RRMS phenotype. In this registry, dimethyl fumarate was prescribed most often (2572/7913, 32.5%), followed by fingolimod (2129/7913, 26.9%) and natalizumab (2021/7913, 25.5%). Details of the study population are shown in Table 1.

**Table 1:** Characteristics of the AMSTR cohort. Data are displayed as n (%) for categorical variables and as median (interquartile range) for continuous variables

AMSTR-cohort: descriptive characteristics including DMT prescription		Total (n = 7913)
Female (%)		5359 (67.7%)
Age baseline (years)		37 (29–45)
Age MS onset * (years)		29 (24–37)
Relapses 12 months prior to baseline †		1 (1–2)
Baseline EDSS ‡		2 (1–3)
EDSS at last FU		2 (1–3.5)
FU duration (months)		44.4 (17.7–90.3)
**Phenotype at first DMT**		
RRMS		7541 (95.4%)
PPMS		226 (2.9%)
SPMS		132 (1.7%)
**Current DMT at end of FU**		
Dimethyl fumarate**		1391 (17.6%)
Fingolimod**		898 (11.3%)
Ofatumumab		766 (9.7%)
Natalizumab		690 (8.7%)
Ocrelizumab		578 (7.3%)
Teriflunomide**		362 (4.6%)
Cladribine		356 (4.5%)
Ozanimod		283 (3.6%)
Siponimod		155 (2.0%)
Ponesimod		150 (1.9%)
Alemtuzumab		41 (0.5%)
**DMT ever patient years on treatment (years)**		
Dimethyl fumarate**	7334.4	2572 (32.5%)
Fingolimod**	10.851.9	2129 (26.9%)
Natalizumab	9556.2	2021 (25.5%)
Ofatumumab	962.9	792 (10.0%)
Teriflunomide**	2609.9	781 (9.9%)
Ocrelizumab	1815	698 (8.8%)
Cladribine	1005.1	458 (5.8%)
Ozanimod	695.2	355 (4.5%)
Siponimod	397.6	200 (2.5%)
Ponesimod	217.3	184 (2.3%)
Alemtuzumab	460.4	90 (1.1%)
Daclizumab	3.3	6 (0.1%)

*AMSTR*  Austrian multiple sclerosis treatment registry, *DMT* disease-modifying therapy, *MS* multiple sclerosis, *EDSS*  expanded disability status scale, *FU* follow up, *RRMS *relapsing–remitting multiple sclerosis, *PPMS* primary-progressive multiple sclerosis, *SPMS* secondary-progressive multiple sclerosis

*Data available for n = 7853

†data available for n = 7906

‡data available for n = 7906

** dimethyl fumarate (n = 9 dimemthylfumarate ratiopharm®), fingolimod (n = 33 fingolimod ratiopharm®, n = 25 fingolimod zentiva®) and teriflunomide (n = 11 Terebyo®) include generic preparations;

This study includes data from pwMS included in the AMSTR who experienced AE (2200/7913, 27.8%). Descriptive information on the AE of each DMT analysed, is presented below. In Table 2***,*** all system organ classes are listed, in which AE for each DMT were reported.

**Table 2:** Distribution of adverse events of the analysed DMTs classified by system organ classes according to the Medical Dictionary for Regulatory Activities (MedDRA)

Adverse events (MedDRA-system organ class)	ALEM (n = 90)		CLAD (n = 458)		DIMET (n = 2572)		FING (n = 2129)		OZAN (n = 355)		PONE (n = 184)		SIPO (n = 200)		NATA (n = 2021)		OCRE (n = 698)		OFAT (n = 792)		TERI (n = 781)	
	n	%	n	%	n	%	n	%	n	%	n	%	n	%	n	%	n	%	n	%	n	%
Infections and infestations	24	26.7	21	4.6	49	1.9	169	7.9	2	0.6	3	1.6	5	2.5	123	6.1	27	3.9	16	2.0	38	4.9
Neoplasms benign, malignant and unspecified (incl cysts and polyps)	0	0.0	3	0.6	9	0.4	31	1.4	3	0.9	0	0.0	3	1.5	11	0.5	2	0.3	2	0.3	2	0.3
Blood and lymphatic system disorders	13	14.4	7	1.5	110	4.3	245	11.5	8	2.3	3	1.6	11	5.5	42	2.1	11	1.6	2	0.3	12	1.5
Immune system disorders	41	45.5	0	0.0	7	0.3	8	0.4	1	0.3	0	0.0	0	0.0	34	1.7	6	0.9	3	0.4	2	0.3
Endocrine disorders	2	2.2	0	0.0	0	0.0	1	0.1	0	0.0	0	0.0	0	0.0	0	0.0	2	0.3	0	0.0	1	0.1
Metabolism and nutrition disorders	1	1.1	0	0.0	8	0.3	1	0.1	1	0.3	0	0.0	0	0.0	1	0.1	0	0.0	0	0.0	0	0.0
Psychiatric disorders	0	0.0	1	0.2	8	0.3	14	0.7	2	0.6	0	0.0	1	0.5	13	0.6	3	0.4	1	0.1	3	0.4
Nervous system disorders	6	6.7	8	1.8	31	1.2	52	2.4	0	0.0	3	1.6	4	2.0	76	3.8	10	1.4	1	0.1	22	2.8
Eye disorders	1	1.1	0	0.0	3	0.1	17	0.8	0	0.0	0	0.0	1	0.5	4	0.2	0	0.0	1	0.1	3	0.4
Ear and labyrinth disorders	0	0.0	0	0.0	1	0.1	2	0.1	0	0.0	0	0.0	0	0.0	1	0.1	0	0.0	0	0.0	1	0.1
Cardiac disorders	2	2.2	0	0.0	5	0.2	35	1.6	0	0.0	1	0.5	2	1.0	6	0.3	0	0.0	0	0.0	3	0.4
Vascular disorders	5	5.5	0	0.0	333	13	9	0.4	7	2.0	0	0.0	0	0.0	9	0.5	1	0.1	1	0.1	36	4.6
Respiratory, thoracic and mediastinal disorders	1	1.1	1	0.2	5	0.2	14	0.7	1	0.3	7	3.8	0	0.0	8	0.4	4	0.6	0	0.0	4	0.5
Gastrointestinal disorders	0	0.0	7	1.5	364	14.2	49	2.3	1	0.3	1	0.5	2	1.0	27	1.3	1	0.1	2	0.3	78	10.0
Hepatobiliary disorders	0	0.0	3	0.7	27	1.1	157	7.4	4	1.1	4	2.2	5	2.5	14	0.7	3	0.4	1	0.1	24	3.1
Skin and subcutaneous tissue disorders	23	25.5	7	1.5	79	3.1	44	2.1	3	0.9	0	0.0	0	0.0	48	2.4	21	3.0	1	0.1	78	10.0
Musculoskeletal and connective tissue disorders	2	2.2	0	0.0	8	0.3	6	0.3	1	0.3	1	0.5	2	1.0	20	1.0	2	0.3	1	0.1	5	0.6
Renal and urinary disorders	2	2.2	0	0.0	2	0.1	8	0.4	0	0.0	0	0.0	0	0.0	4	0.2	1	0.1	0	0.0	2	0.3
Pregnancy, puerperium and perinatal conditions	2	2.2	2	0.4	0	0.0	2	0.1	0	0.0	0	0.0	0	0.0	0	0.0	0	0.0	0	0.0	1	0.1
Reproductive system and breast disorders	1	1.1	1	0.2	4	0.2	5	0.2	0	0.0	0	0.0	0	0.0	2	0.1	0	0.0	0	0.0	5	0.6
General disorders and administration site conditions	6	6.7	5	1.1	29	1.1	35	1.6	0	0.0	0	0.0	0	0.0	65	3.2	7	1.0	0	0.0	9	1.2
Investigations	0	0.0	0	0.0	8	0.3	5	0.2	0	0.0	0	0.0	0	0.0	11	0.5	0	0.0	0	0.0	2	0.3
Injury, poisoning and procedural complications	4	4.4	0	0.0	0	0.0	0	0.0	0	0.0	0	0.0	0	0.0	19	1	1	0.1	0	0.0	0	0.0
unkown	0	0.0	0	0.0	0	0.0	1	0.1	0	0.0	0	0.0	0	0.0	0	0.0	0	0.0	0	0.0	1	0.1

### Alemtuzumab

Out of 90 patients who were treated with alemtuzumab, 63 (70%) were reported to have experienced AE. Among these, 41/90 (45.5%) subjects had AE involving the immune system, including autoimmune diseases affecting the thyroid gland (24/90, 26.7%) and allergic reactions (16/90, 17.8%). Twenty four out of 90 (26.7%) patients had infections documented in the AMSTR during the treatment with alemtuzumab. The most common infectious diseases were influenza or influenza-like illnesses (9/90, 10%), herpes (8/90, 8.9%) as well as urinary tract infections (2/90, 2.2%). The skin was the third most common organ affected (23/90, 25.5%), with rash being the most common complaint (13/90, 14.4%). Four patients (4/90, 4.4%) experienced IRR. One patient (1/90, 1.1%) suffered from hemophagocytic lymphohistiocytosis.

Out of 90 patients, four (4.4%) experienced SAE. Among them, two (2.2%) suffered from allergic reactions requiring medical intervention. An ischemic stroke and the detection of tuberculosis, indicated by increased interferon-gamma, each occurred in a single patient, respectively. Three patients (3.3%) reported the following AE at discontinuation: hyperthyroidism, autoimmune thyroiditis, and fatigue.

### Cladribine

In total, 35/458 (7.6%) patients were reported to have experienced AE while being treated with cladribine. Infections appeared in 21 (4.6%) patients with herpes (10/458, 2.2%), urinary tract infection (4/458, 0.9%) and influenza or influenza-like illness (2/458, 0.4%) being the most frequent ones. The second most common AE affected the nervous system, with 3 (0.7%) of the 8 (1.8%) reported AEs being headaches, 2 (0.4%) being vertigo, and 1 (0.2%) being a stroke. Regarding the blood system, an AE was reported in 7 (1.8%) patients, with 3 (0.7%) having lymphopenia and 2 (0.4%) having leukopenia.

In total, 5 patients (1.1%) experienced the following SAE: 3 patients had a neoplasia (3/458, 0.7%) of which one patient developed a melanoma, one a Mycosis fungoides and one a neoplasia without specific information. One had an ischemic stroke, and one patient had a spontaneous abortion (1/458, 0.2% each). The following AE were reported at the time of treatment discontinuation in two patients (0.4%): lymphopenia in one case and vertigo and dry skin in the other.

### Dimethyl fumarate

Out of 2572 patients receiving dimethyl fumarate, 733 (28.6%) were reported to have experienced AE. In total, 364 patients (14.2%) suffered from GI-AE, such as diarrhoea (93/2572, 3.6%), abdominal pain (66/2572, 2.6%), nausea (57/2572, 2.2%), and GI-cramps (25/2572, 1.0%). Vascular AE occurred in more than one-tenth of the patients (333/2572, 13%). All but one of these patients had flushing symptoms (13%). Overall, 49/2572 (1.9%) pwMS reported infections during their therapy. The most common infections were influenza, influenza-like illnesses or coronavirus disease 2019 (COVID-19; 17/2572, 0.7%), herpes (15/2572, 0.6%), and pneumonia (4/2572, 0.2%).

Thirteen patients (13/2572, 0.5%) had SAE during the treatment with dimethyl fumarate. Neoplasms were the most common SAE, affecting 9 patients (9/2572, 0.4%). The following neoplasms were reported: melanoma (2/2572, 0.1%), breast carcinoma (2/2572, 0.1%), tonsillar carcinoma (1/2572, < 0.1%), meningioma (1/2572, < 0.1%), basal cell carcinoma (1/2572, < 0.1%), one of MedDRA category 20 without further specification (1/2572, < 0.1%) and one patient was found to have an inguinal nodule for which no histologic work up was available (1/2572, < 0.1%). The other SAE included anaphylactic shock (1/2572, < 0.1%), suicidality in known bipolar disorder (1/2572, < 0.1%), varicella zoster virus meningitis (1/2672, < 0.1%) and ischemic stroke (1/2572, < 0.1%). At discontinuation of dimethyl fumarate therapy AE were reported in 418/2572 (16.3%) patients. Most of these pwMS suffered from AE affecting their GI-tract (171/2572, 6.7%), the blood system (94/2572, 3.7%), and the skin (78/2572, 3.0%).

### Fingolimod

From 2129 patients treated with fingolimod, 615 (28.9%) were reported to have an AE. The most common AE reported to have occurred during fingolimod treatment affected the blood system (245/2129, 11.5%). Among them, 170/2129 (8%) had a reported lymphopenia, and one of them presented with atypical lymphocytes (1/2129, 0.1%). Other 42/2129 (2%) subjects suffered from leukopenia. The second most common AE were infections: 169/2129 (7.9%). Influenza, influenza-like illnesses or COVID-19 were reported in 49/2129 (2.3%) patients, 40/2129 (1.9%) had herpes infection (27 herpes zoster, 13 herpes simplex), 12/2129 (0.6%) had urinary tract infections, and 8/2129 (0.4%) had pneumonia. A total of 157/2129 (7.4%) patients had AE affecting the hepatobiliary system, and 125/2129 (5.9%) showed elevated hepatic enzyme levels. AE affecting the cardiac system were observed in 35/2129 (1.6%) patients, including 5/2129 (0.2%) who developed atrioventricular (AV) block and 11/2129 (0.5%) who developed bradycardia. Furthermore, 14/2129 (0.7%) pwMS suffered from tachycardia or palpitations.

Approximately 2% (43/2129) of the subjects treated with fingolimod suffered from SAE. Most of these SAE were neoplasms observed in 31/2129 (1.4%) patients. Among them, 13/2129 (0.6%) developed basal cell carcinoma, 5/2129 (0.2%) were diagnosed with melanoma, 2/2129 (< 0.1%) had a cervix dysplasia, 1/2129 (< 0.1%) patient each had a verrucous squamous cell carcinoma, a bronchial carcinoma, a T-cell lymphoma, a mamma carcinoma, a papillary thyroid carcinoma, a polyp and a cyst. For four subjects details on the type of neoplasm were not documented. The following infectious diseases were diagnosed in four patients (4/2129, 0.2%): zoster meningitis, acute respiratory distress syndrome triggered by pneumonia, severe sepsis and urosepsis (1/2129, < 0.1% each). One subject each developed AV block III and endocarditis, and two patients (0.1%) had AE related to their pregnancy (ectopic pregnancy and spontaneous abortion). One patient each experienced pancreatitis, anaphylactic shock, an ischemic stroke, and hepatitis. In total, 298/2129 (14.0%) reported AE at discontinuation of therapy with fingolimod. Most of them reported AE affecting the blood system (94/2129, 4.4%), hepatobiliary system (64/2129, 3.0%), or had infections (38/2129, 1.8%).

### Ozanimod

Of a total of 355 patients treated with ozanimod, 32 (9%) reported AE. The most commonly reported AE affected the blood system (8/355, 2.3%), with lymphopenia (6/355, 1.7%) being the most frequent one. Vascular AE (7/355, 2%) were the second most frequently reported AE, with hypertension (6/355, 1.7%) being most common.

Of note, 3 SAE (0.9%) were also reported, consisting of one melanoma, one basal cell carcinoma and one cervical intraepithelial neoplasia – CIN II (1/355, 0.3% each).

Thirteen out of 355 (3.7%) patients reported AE at the time of treatment discontinuation. Most of them affected the blood system (4/355, 1.1%), the hepatobiliary system (4/355, 1.1%) and the skin (2/355, 0.6%). In addition, one patient had depression, one respiratory tract AE, and one disorders of the vascular and immune systems at time of discontinuation.

### Ponesimod

AE were recorded in 21/184 (11.4%) patients treated with Ponesimod. The most frequently reported AE were those affecting the respiratory tract (7/184, 3.8%), with two cases of dyspnea (1.1%), which were also classified as SAE. This was followed by AE affecting the hepatobiliary system (4/184, 2.2%), with three reported cases (1.6%) of elevated hepatic enzyme levels.

Thirteen patients (13/184, 7.1%) reported AE when discontinuing treatment with ponesimod. Collected AE at that time related to respiratory tract disorders (4/184, 2.2%), blood system disorders (3/184, 1.6%), nervous system disorders (3/184, 1.6%), and hepatobiliary AE (2/184, 1.1%), with one of the latter also experiencing urinary tract infections, and one patient (0.5%) experiencing palpitations.

### Siponimod

Among patients treated with siponimod, 31/200 (15.5%) reported AE. The most common AE were those affecting the blood system (11/200, 5.5%), with lymphopenia (9/200, 4.5%) being the most common one. This was followed by AE related to the hepatobiliary system (5/200, 2.5%), with elevated hepatic enzyme levels (4/200, 2%) being the most common.

In terms of SAE, 3 patients had neoplasms (1.5%). These included one melanoma, one basal cell carcinoma, and one squamous cell carcinoma of the skin (1/200, 0.5% each). In total, 17/200 (8.5%) patients reported AE at discontinuation of treatment with siponimod. These AE most frequently affected the blood system (7/200, 3.5%), the hepatobiliary system (4/200, 2.0%) or headaches (3/200, 1.5%).

### Natalizumab

In total, 342/2021 (16.9%) of patients who were treated with natalizumab were reported to have experienced AE. The most common AE were infections, which were reported in 123/2021 (6.1%) patients, such as influenza, influenza-like illnesses or COVID-19 (34/2021, 1.7%), herpes infection (28/2021, 1.4%), urinary tract infections (15/2021, 0.7%), upper respiratory tract and ear infections (12/2021, 0.6%), and pneumonia (5/2021, 0.3%). AE affecting the nervous system were reported in 76/2021 (3.8%) patients. These AEs include headaches (40/2021, 2%) and dizziness (13/2021, 0.6%). Furthermore, 69/2021 (3.4%) suffered from other symptoms, like fatigue (34/2021, 1.7%), influenza-like symptoms (5/2021, 0.2%), impaired concentration (4/2021, 0.2%), and oedema (3/2021, 0.2%). Twenty patients (1%) experienced an IRR.

SAE were observed in 28/2021 (1.3%) patients. The most common SAE were neoplasms, which were diagnosed in 11/2021 patients (0.5%), and included the following: seminoma (2/2021, 0.1%), cervical cancer (2/2021, 0.1%), breast carcinoma (2/2021, 0.1%), pancreas carcinoma (1/2021, 0.1%), gastric carcinoma (1/2021, 0.1%), Glioma WHO I-II (1/2021, 0.1%), pituitary adenoma (1/2021, 0.1%) and uterine fibroid (1/2021, 0.1%). The second most common SAE were severe infections (10/2021, 0.5%), including verified PML (7/2021, 0.3%), osteomyelitis (1/2021, 0.1%), severe pneumonia that resulted in hospitalization (1/2021, 0.1%) and urosepsis (1/2021, 0.1%). Furthermore, 4/2021 patients (0.2%) had allergic reactions requiring medical intervention, one had a sinus venous thrombosis (1/2021, 0.1%) and 1/2021 (0.1%) patient each experienced dyspnea and bronchospasm. In total, 134/2021 (6.6%) patients reported AE at treatment discontinuation. These AE affected mostly the following system organ classes: infections (35/2021, 1.7%), the immune system (23/2021, 1.1%), and the nervous system (15/2021, 0.7%).

### Ocrelizumab

Out of 698 patients who received therapy with ocrelizumab, 83 pwMS (11.9%) were reported to have suffered from AE. Most patients had infections (27/698, 3.9%). The most common infection were influenza, influenza-like illnesses or COVID-19 (9/698, 1.3%) and herpes (3/698, 0.4%). Twenty-one patients (21/698, 3%) had AE that affected their skin, with Exanthem in 10/698 (1.4%) being the most frequent one. Six pwMS suffered from pruritus (0.9%) as IRR and two patients each (0.3%) developed a rash and folliculitis.

Three patients (0.4%) suffered from SAE. One patient was diagnosed with a glioblastoma and one with a rectal carcinoma (1/698, 0.1% each). Another patient experienced dyspnea (1/698, 0.1%). In total, 21 of 698 patients (3.0%) reported AE at treatment discontinuation. The most frequent AE related to the blood and lymphatic system (4/698, 0.6%), the skin (4/698, 0.6%), psychiatric disorders (3/698, 0.4%), and infections (2/698, 0.3%).

### Ofatumumab

A total of 792 patients received Ofatumumab, with 35 (4.4%) patients reporting an AE. The most common AE were infections, which were reported in 16/792 (2%) patients, such as herpes infections (6/792, 0.8%), upper respiratory tract infections (3/792, 0.4%), pneumonia (2/792, 0.3%) and urinary tract infections (2/792, 0.3%).

A total of 2 SAE (2/792, 0.2%) were reported. One (0.1%) pituitary adenoma and one (0.1%) mammary duct papilloma. Overall, from 7/792 (0.9%) patients AE data were registered at treatment discontinuation, most commonly infections (2/792, 0.3%) and immune system disorders (2/792, 0.3%) with one patient having both (1/792, 0.1%).

### Teriflunomide

With 781 patients on teriflunomide, 210 (27%) reported AE. The most common AE were skin-related (79/781, 10.1%), with the majority (60/781, 7.7%) suffering from alopecia. 0ne in ten patients treated with teriflunomide experienced GI-tract related AE (78/781, 10%). Among them, the majority of pwMS (38/781, 4.9%) suffered from diarrhea. Six of 781 (0.8%) patients suffered from inflammation of different parts of the GI-tract. A total of 38/781 (4.9%) patients experienced infections while taking teriflunomide, with the most common infections being influenza or influenza-like illnesses (9/781, 1.2%), followed by herpes virus infections (8/781, 1%), infections of the upper respiratory tract and ears (5/781, 0.6%), and GI-infections (5/781, 0.6%). Furthermore, 36/781 (4.6%) pwMS suffered from hypertension.

Overall, 6/781 (0.8%) patients suffered from SAE. One patient (0.1%) had a dysplastic nevus. One patient (0.1%) experienced recurrent dyspnea and thoracic pressure, one (0.1%) had an endometrial polyp, one (0.1%) had a liver failure, one a pancolitis caused by cytomegalovirus and one (0.1%) a spondylodiscitis with iliopsoas abscess. Of the 781 patients documented in the registry, 109 (14%) reported AE when discontinuing therapy with teriflunomide. The majority of these AE affected the GI-tract (48/781, 6.2%), skin (15/781, 1.9%), or hepatobiliary system (18/781, 2.3%).

### Other DMT

No AE were documented in the registry for patients under Daclizumab (n = 6). Of note, the case number is low and the follow up period was relatively short.

ALEM: Alemtuzumab, CLAD: Cladribine, DIMET: dimethyl fumarate, FING: fingolimod, NATA: Natalizumab, OCRE: Ocrelizumab, OFAT: Ofatumumab, OZAN: Ozanimod, PONE: Ponesimod, SIPO: Siponimod, TERI: Teriflunomide;

In addition, the supplements contain a list of AE reported for each DMT with a comparison of the respective frequencies according to the package insert. Furthermore, SAE of the respective DMT are summarized in Table 3.

**Table 3:** DMT and reported SAE with their respective case numbers (%) for overall, neoplasia, infections and pregnancy-related SAE and all other SAE summarized under others

DMT	SAE				
	Overall	neoplasia	infections	pregnancy-related	others
Alemtuzumab (n = 90)	4 (4.4%)	0 (0%)	0 (0%)	0 (0%)	4 (4.4%)
Cladribine (n = 458)	5 (1.1%)	3 (0.7%)	0 (0%)	1 (0.2%)	1 (0.2%)
Dimethyl fumarate (n = 2567)	13 (0.5%)	9 (0.4%)	1 (< 0.1%)	0 (0%)	3 (0.1%)
Fingolimod (n = 2128)	43 (2%)	31 (1.4%)	4 (0.2%)	2 (0.1%)	6 (0.3%)
Ozanimod (n = 355)	3 (0.9%)	3 (0.9%)	0 (0%)	0 (0%)	0 (0%)
Ponesimod (n = 184)	2 (1.1%)	0 (0%)	0 (0%)	0 (0%)	2 (1.1%)
Siponimod (n = 200)	3 (1.5%)	3 (1.5%)	0 (0%)	0 (0%)	0 (0%)
Natalizumab (n = 2021)	28 (1.4%)	11 (0.5%)	10 (0.5%)	0 (0%)	7 (0.4%)
Ocrelizumab (n = 698)	2 (0.8%)	1 (0.4%)	0 (0%)	0 (0%)	1 (0.4%)
Ofatumumab (n = 792)	2 (0.2%)	2 (0.2%)	0 (0%)	0 (0%)	0 (0%)
Teriflunomide (n = 777)	3 (0.6%)	2 (0.3%)	0 (0%)	0 (0%)	2 (0.3%)

*DMT* disease modifying therapy, *SAE* severe adverse event

## Discussion

In this retrospective analysis of the AMSTR, safety data from approximately 8000 MS patients treated with several DMT within an observation period of 19 years were analyzed. The reported AE aligned with the known safety profiles of the DMT, with no evidence of increased AE rates or the emergence of new, drug-specific AE patterns compared to approval and post-marketing data.

Immune-related AE, particularly thyroid disorders, were most common with alemtuzumab. Infections predominated among patients treated with cladribine, natalizumab, ocrelizumab and ofatumumab. Gastrointestinal side effects were frequently observed with dimethyl fumarate and teriflunomide, while sphingosine-1-phosphate modulators (S1P-modulators; fingolimod, ozanimod, ponesimod, siponimod) were associated primarily with blood system-related AE. These findings suggest consistent safety outcomes across the different DMT [10, 12–22].

Examining the SAE in greater depth, alemtuzumab in particular showed a high SAE rate of 4.4% in addition to the high AE rate of 70%. This has already been investigated in detail by the European Medicines Agency (EMA) and resulted in an updated limited use authorization for adult patients with rapidly evolving severe RRMS or inadequate response to high-efficacy DMT, and requires rigorous safety monitoring [23]. Apart from this, for all other DMT low SAE rates were reported. In particular, the neoplasia rates were low for all DMT. Notably, S1P-modulators showed the highest neoplasia rates (fingolimod 31/2129, 1.4%, siponimod 3/200, 1.5%), whereby basal cell carcinomas accounted for the largest proportion (fingolimod 13/31, 42%; siponimod 1/3, 33.3%). However, neoplasia numbers largely align with expected rates observed in matched reference populations [13, 14, 19, 21, 22, 24]. Moreover, data on a borderline-significant increased risk of cancer were published specifically for fingolimod, though the risk was not attributable to a specific type of cancer [25]. One possible explanation for the increased risk of cancer with S1P-R modulators is that the number of circulating immune cells is reduced, putatively limiting the ability to recognize and eliminate malignant cells [26]. In addition, the product information from EMA refers to a possible risk of cutaneous malignancies and lymphomas which is why at initiation and every 6–12 months thereafter, a dermatological screening examination is recommended as part of DMT monitoring [27]. Notably, general standard screening procedures for cancer should be followed for all pwMS.

With respect to SAE, only for fingolimod (4/2129, 0.2%), natalizumab (10/2021, 0.5%) and dimethyl fumarate (1/2572, < 0.1%) severe infections were reported, albeit rarely. Notably, there was one case of zoster meningitis, but no cases of cryptococcosis regarding fingolimod. For natalizumab, 7 confirmed progressive multifocal leukoencephalopathy (PML) cases (0.3%) were reported. This is comparable to the data from other large surveillance programs [10]. Additionally, reference is made here to the risk stratification model published in 2017 [28].

Pregnancy-related SAE were only reported for cladribine (1/458, 0.2%) and fingolimod (2/2129, 0.1%). In a limited analysis of 70 pregnancies under cladribine treatment, the pregnancy outcomes, including spontaneous abortion, were comparable to the general population [29, 30]. Nevertheless, according to the product information from EMA, effective contraception during cladribine treatment and for at least 6 months after the last dose is recommended in both female and male pwMS [31]. It is important to note that, according to a statement by the EMA, fingolimod is associated with a two-fold increased risk of congenital malformations in infants exposed in utero, compared to the baseline risk of 2–3% in the general population [32]. Thus, an effective contraception for women taking fingolimod is recommended during DMT intake and until 2 months after discontinuation of treatment with fingolimod [32].

### Limitations

This study clearly has limitations. First of all, the retrospective nature should be mentioned, although therapy-registries with respect to real-world represent a crucial pillar of available evidence. Secondly, the case numbers for the different DMT differ significantly, which is explained by the observation period and the different approval dates of the respective DMT. Thirdly, it is important to acknowledge that AE recorded in the registry are limited to those entered by treating neurologists, which may depend on individual clinical judgment – for example, whether to report laboratory abnormalities as AE. It should also be noted that the reported AE at the time of treatment discontinuation do not necessarily represent the reason for discontinuation. Nevertheless, the overall reported rates of AE and SAE are largely consistent with existing published data. With regard to reporting bias, it should be noted that the AMSTR includes data on 7913 pwMS, meaning that not all individuals receiving DMTs in Austria are represented. This is partly due to the fact that not all DMTs are captured in the AMSTR (e.g. interferon-beta preparations and glatirameracetat). Last but not least, the study cohort mainly consisted of patients of Caucasian origin, which restricts the generalizability of the data for other ethnicities.

## Conclusion

In conclusion, the safety data of the AMSTR are consistent with previously published data on respective DMT and raise no new safety issues. Notably, use of alemtuzumab is limited by the EMA to adult patients with rapidly evolving severe RRMS or inadequate response to high-efficacy DMT, and requires rigorous safety monitoring. Our results clearly emphasize the need for (nation-wide) registries to generate data on the long-term safety of new DMT. To date, no significant differences have been observed – particularly regarding the risk of infections and neoplasms – between the pivotal phase 3 clinical trials and real-world data on the use of these DMT. This consistency in safety outcomes provides reassurance for both pwMS and treating neurologists, supporting the continued use of these therapies in routine clinical practice.

## Supplementary Information

Below is the link to the electronic supplementary material.

Supplementary file1 (DOCX 36 KB)

## References

[1] Walton C, King R, Rechtman L et al (2020) Rising prevalence of multiple sclerosis worldwide: insights from the atlas of MS, third edition. Mult Scler J 26:1816–1821. 10.1177/1352458520970841 10.1177/1352458520970841 PMC7720355 33174475

[2] Rae-Grant A, Day GS, Marrie RA et al (2018) Practice guideline recommendations summary: disease-modifying therapies for adults with multiple sclerosis. Neurology 90:777–788 29686116 10.1212/WNL.0000000000005347

[3] Hauser SL, Cree BAC (2020) Treatment of multiple sclerosis: a review. Am J Med 133:1380-1390.e2 32682869 10.1016/j.amjmed.2020.05.049 PMC7704606

[4] He A, Merkel B, Brown JWL et al (2020) Timing of high-efficacy therapy for multiple sclerosis: a retrospective observational cohort study. Lancet Neurol 19:307–316. 10.1016/S1474-4422(20)30067-3 32199096 10.1016/S1474-4422(20)30067-3

[5] Winkelmann A, Loebermann M, Reisinger EC et al (2016) Disease-modifying therapies and infectious risks in multiple sclerosis. Nat Rev Neurol 12:217–233 26943779 10.1038/nrneurol.2016.21

[6] Melamed E, Lee MW (2020) Multiple sclerosis and cancer: the Ying-Yang effect of disease modifying therapies. Front Immunol. 10:497041 10.3389/fimmu.2019.02954 PMC6965059 31998289

[7] Berger T, Kobelt G, Berg J et al (2017) New insights into the burden and costs of multiple sclerosis in Europe: results for Austria. Mult Scler J 23:17–28. 10.1177/1352458517708099 10.1177/1352458517708099 28643599

[8] Berger T, Adamczyk-Sowa M, Csépány T et al (2018) Management of multiple sclerosis patients in central European countries: current needs and potential solutions. Ther Adv Neurol Disord. 10.1177/1756286418759189 29511382 10.1177/1756286418759189 PMC5826096

[9] Guger M, Enzinger C, Leutmezer F et al (2019) Real-life use of oral disease-modifying treatments in Austria. Acta Neurol Scand 140:32–39. 10.1111/ane.13097 30958901 10.1111/ane.13097 PMC6767158

[10] Monschein T, Dekany S, Zrzavy T et al (2023) Real-world use of natalizumab in Austria: data from the Austrian Multiple Sclerosis Treatment Registry (AMSTR). J Neurol. 10.1007/s00415-023-11686-2 37074388 10.1007/s00415-023-11686-2 PMC10344989

[11] Guger M, Enzinger C, Leutmezer F et al (2021) Long-term outcome and predictors of long-term disease activity in natalizumab-treated patients with multiple sclerosis: real life data from the Austrian MS treatment registry. J Neurol 268:4303–4310. 10.1007/s00415-021-10559-w 33890167 10.1007/s00415-021-10559-w PMC8505366

[12] Ziemssen T, Bass AD, Berkovich R et al (2020) Efficacy and safety of alemtuzumab through 9 years of follow-up in patients with highly active disease: post hoc analysis of CARE-MS I and II patients in the TOPAZ extension study. CNS Drugs. 10.1007/s40263-020-00749-x 32710396 10.1007/s40263-020-00749-x PMC7447657

[13] Steingo B, Al Malik Y, Bass AD et al (2020) Long-term efficacy and safety of alemtuzumab in patients with RRMS: 12-year follow-up of CAMMS223. J Neurol. 10.1007/s00415-020-09983-1 32583052 10.1007/s00415-020-09983-1 PMC7578137

[14] Leist T, Cook S, Comi G et al (2020) Long-term safety data from the cladribine tablets clinical development program in multiple sclerosis. Mult Scler Relat Disord. 10.1016/j.msard.2020.102572 33296971 10.1016/j.msard.2020.102572

[15] Giovannoni G, Soelberg Sorensen P, Cook S et al (2018) Safety and efficacy of cladribine tablets in patients with relapsing–remitting multiple sclerosis: results from the randomized extension trial of the CLARITY study. Mult Scler J. 10.1177/1352458517727603 10.1177/1352458517727603 28870107

[16] Gold R, Comi G, Palace J et al (2014) Assessment of cardiac safety during fingolimod treatment initiation in a real-world relapsing multiple sclerosis population: a phase 3b, open-label study. J Neurol. 10.1007/s00415-013-7115-8 24221641 10.1007/s00415-013-7115-8 PMC3915082

[17] Cohen JA, Khatri B, Barkhof F et al (2016) Long-term (up to 4.5 years) treatment with fingolimod in multiple sclerosis: results from the extension of the randomised TRANSFORMS study. J Neurol Neurosurg Psychiatry. 10.1136/jnnp-2015-310597 26111826 10.1136/jnnp-2015-310597 PMC4853559

[18] Rudick RA, Panzara MA (2008) Natalizumab for the treatment of relapsing multiple sclerosis. Biologics. 10.2147/btt.s1956 19707353 10.2147/btt.s1956 PMC2721353

[19] Hauser SL, Kappos L, Montalban X et al (2021) Safety of Ocrelizumab in patients with relapsing and primary progressive multiple sclerosis. Neurology. 10.1212/WNL.0000000000012700 34475123 10.1212/WNL.0000000000012700 PMC8548959

[20] Miller AE, Olsson TP, Wolinsky JS et al (2020) Long-term safety and efficacy of teriflunomide in patients with relapsing multiple sclerosis: results from the TOWER extension study. Mult Scler Relat Disord. 10.1016/j.msard.2020.102438 32911306 10.1016/j.msard.2020.102438

[21] O’Connor P, Comi G, Freedman MS et al (2016) Teriflunomide Multiple Sclerosis Oral (TEMSO) Trial Group and the MRI-AC in Houston Texas. Long-term safety and efficacy of teriflunomide: Nine-year follow-up of the randomized TEMSO study. Neurology. 86(10):920–30. 10.1212/WNL.0000000000002441 26865517 10.1212/WNL.0000000000002441 PMC4782117

[22] Gold R, Arnold DL, Bar-Or A et al (2022) Long-term safety and efficacy of dimethyl fumarate for up to 13 years in patients with relapsing-remitting multiple sclerosis: final ENDORSE study results. Mult Scler J. 10.1177/13524585211037909 10.1177/13524585211037909 PMC8978463 34465252

[23] https://www.ema.europa.eu/en/medicines/human/referrals/lemtrada.

[24] Nasirzadeh A, Jahanshahi R, Ghajarzadeh M et al (2023) The prevalence of cancer in patients with multiple sclerosis (MS) who were Under treatment with Natalizumab (Tysabri): a systematic review and meta-analysis. Int J Prev Med. 14(1):114 38264560 10.4103/ijpvm.ijpvm_77_22 PMC10803669

[25] Alping P, Askling J, Burman J et al (2020) Cancer risk for Fingolimod, Natalizumab, and Rituximab in multiple sclerosis patients. Ann Neurol. 10.1002/ana.25701 32056253 10.1002/ana.25701

[26] Killestein J, Leurs CE, Hoogervorst ELJ et al (2017) Clinical/scientific notes five cases of malignant melanoma during fingolimod treatment in dutch patients with MS. Published Online First. 10.1136/bcr 10.1212/WNL.0000000000004293 28768850

[27] https://www.ema.europa.eu/en/medicines/human/EPAR/gilenya#product-info.

[28] Ho PR, Koendgen H, Campbell N et al (2017) Risk of natalizumab-associated progressive multifocal leukoencephalopathy in patients with multiple sclerosis: a retrospective analysis of data from four clinical studies. Lancet Neurol 16:925–933. 10.1016/S1474-4422(17)30282-X 28969984 10.1016/S1474-4422(17)30282-X

[29] Giovannoni G, Galazka A, Schick R et al (2020) Pregnancy outcomes during the clinical development program of cladribine in multiple sclerosis: an integrated analysis of safety. Drug Saf. 10.1007/s40264-020-00948-x 32447743 10.1007/s40264-020-00948-x PMC7305061

[30] Dost-Kovalsky K, Thiel S, Ciplea AI et al (2023) Cladribine and pregnancy in women with multiple sclerosis: the first cohort study. Mult Scler J. 10.1177/13524585221131486 10.1177/13524585221131486 36278327

[31] https://www.ema.europa.eu/en/medicines/human/EPAR/mavenclad#product-info.

[32] https://www.ema.europa.eu/en/news/updated-restrictions-gilenya-multiple-sclerosis-medicine-not-be-used-pregnancy.

