# Supplementary material for: Safety of disease-modifying therapies in multiple sclerosis: real-world data from the Austrian MS Treatment Registry (AMSTR)
Source: J Neurol. 2025 Nov 19;272(12):774. doi: 10.1007/s00415-025-13473-7 (PMC12630275; doi:10.1007/s00415-025-13473-7)
Supplement: Supplementary file 1 — Supplementary file1 (DOCX 36 KB) [file 415_2025_13473_MOESM1_ESM.docx]

**Supplement**

| **Alemtuzumab** (frequency according to product information) | **Adverse events** | **n** | **%** |
| --- | --- | --- | --- |
| Very common (>1/10) | Headache | 1 | 1.11 |
|  | Fatigue | 1 | 1.11 |
|  | Neutropenia | 1 | 1.11 |
|  | Pruritus | 1 | 1.11 |
|  | Tachycardia | 1 | 1.11 |
|  | Chills | 2 | 2.22 |
|  | Leukopenia | 2 | 2.22 |
|  | Urinary tract infection | 2 | 2.22 |
|  | Lymphopenia | 4 | 4.44 |
|  | Herpes infection | 10 | 11.11 |
|  | Upper respiratory tract infection | 10 | 11.11 |
|  | Basedow’s disease, hyperthyrodisim, hypothyroidism | 12 | 13.33 |
|  | Rash | 13 | 14.44 |
| Common  (≥1/100 to <1/10) | Vision impairment | 1 | 1.11 |
|  | Acne | 1 | 1.11 |
|  | Menstruation irregular | 1 | 1.11 |
|  | Alopecia | 1 | 1.11 |
|  | Skin lesion | 1 | 1.11 |
|  | Erythema | 1 | 1.11 |
|  | Hypertension | 2 | 2.22 |
|  | Myalgia, arthralgia | 2 | 2.22 |
|  | Thrombocytopenia | 3 | 3.33 |
|  | Influenza -like symptoms | 4 | 4.44 |
|  | Autoimmune thyroiditis including thyroiditis subacute | 12 | 13.33 |
|  | Allergic reaction | 16 | 17.77 |
| Uncommon  (≥1/1,000 to <1/100) | Tuberculosis | 1 | 1.11 |
| rare (≥1/10,000 to <1/1,000) | Haemophagocytic lymphohistiocytosis | 1 | 1.11 |
| Not mentioned in the product information | Fever | 1 | 1.11 |
|  | Photophobia | 1 | 1.11 |
|  | Ischemic event | 1 | 1.11 |
|  | Hematuria | 1 | 1.11 |
|  | Bradycardia | 1 | 1.11 |
|  | Dysnpoea | 1 | 1.11 |
|  | Erythema | 4 | 4.44 |

Supplementary Table 1: Frequency of AE with alemtuzumab in the AMSTR cohort compared with the frequency stated in the product information.

| **Dimethyl fumarate** (frequency according to product information) | **Adverse events** | **n** | **%** |
| --- | --- | --- | --- |
| Very common (>1/10) | Nausea | 57 | 2.21 |
|  | Abdominal pain | 66 | 2.56 |
|  | Diarrhoea | 93 | 3.61 |
|  | Flush | 332 | 12.90 |
| Common  (≥1/100 to <1/10) | Gastritis | 9 | 0.34 |
|  | Erythema | 10 | 0.38 |
|  | Feeling hot | 12 | 0.46 |
|  | Rash | 13 | 0.50 |
|  | Alopecia | 16 | 0.62 |
|  | ALT/AST increase | 21 | 0.81 |
|  | Leucopenia | 21 | 0.81 |
|  | Pruritus | 32 | 1.24 |
|  | Lymphopenia | 88 | 3.42 |
| Uncommon  (≥1/1,000 to <1/100) | Thrombocytopenia | 2 | 0.13 |
|  | Allergic reaction | 6 | 0.23 |
| Not mentioned in the product information | Fatigue | 3 | 0.11 |
|  | Urinary tract infection | 3 | 0.11 |
|  | Headache | 4 | 0.15 |
|  | Arthralgia | 4 | 0.15 |
|  | Dizziness | 4 | 0.15 |
|  | Pneumonia | 4 | 0.15 |
|  | Loose stool | 5 | 0.19 |
|  | Meteorism | 6 | 0.23 |
|  | Constipation | 7 | 0.27 |
|  | Weight loss | 9 | 0.34 |
|  | Dysesthesia | 9 | 0.34 |
|  | Herpes infection | 15 | 0.58 |
|  | Muscle cramps | 15 | 0.58 |
|  | Influenza or influenza-like illnesses | 17 | 0.66 |

Supplementary Table 2: Frequency of AE with dimethyl fumarate in the AMSTR cohort compared with the frequency stated in the product information. AE with a relative frequency <0.1% that are not mentioned in the product information are not shown in the table.

| **Fingolimod** (frequency according to product information) | **Adverse events** | **n** | **%** |
| --- | --- | --- | --- |
| Very common (>1/10) | Sinusitis | 5 | 0.23 |
|  | Basal cell carcinoma | 13 | 0.61 |
|  | Diarrhoea | 14 | 0.65 |
|  | Headache | 19 | 0.89 |
|  | Influenza or influenza-like illnesses | 49 | 2.30 |
|  | Hepatic enzyme increased | 129 | 6.05 |
| Common  (≥1/100 to <1/10) | Migraine | 3 | 0.14 |
|  | Myalgia, arthralgia | 4 | 0.18 |
|  | Bronchitis | 5 | 0.23 |
|  | Atrioventricular block | 5 | 0.23 |
|  | Fever | 7 | 0.32 |
|  | Depression | 7 | 0.32 |
|  | Hypertension | 8 | 0.37 |
|  | Bradycardia | 11 | 0.51 |
|  | Dizziness | 12 | 0.56 |
|  | Alopecia | 15 | 0.70 |
|  | Herpes infection | 40 | 1.87 |
|  | Leukopenia | 42 | 1.97 |
|  | Lymphopenia | 170 | 7.98 |
| Uncommon  (≥1/1,000 to <1/100) | Melanoma | 5 | 0.23 |
|  | Pneumonia | 8 | 0.37 |
|  | Macular oedema | 9 | 0.42 |
|  | Nausea | 9 | 0.46 |
| Not mentioned in the product information | Mycosis | 3 | 0.14 |
|  | Anemia | 3 | 0.14 |
|  | Granulopenia | 3 | 0.14 |
|  | Aphthae | 3 | 0.14 |
|  | Conjunctivitis | 3 | 0.14 |
|  | Anxiety | 3 | 0.14 |
|  | Abdominal pain | 5 | 0.23 |
|  | Allergic reaction | 5 | 0.23 |
|  | Tonsillitis/pharyngitis/otitis | 5 | 0.23 |
|  | Warts | 6 | 0.28 |
|  | Exanthema | 6 | 0.28 |
|  | Gastrointestinal infection | 6 | 0.28 |
|  | Gastrointestinal inflammation | 7 | 0.32 |
|  | Urinary tract infection | 12 | 0.56 |
|  | Fatigue | 13 | 0.61 |
|  | Palpitations or tachycardia | 14 | 0.61 |

Supplementary Table 3: Frequency of AE with fingolimod in the AMSTR cohort compared with the frequency stated in the product information. AE with a relative frequency <0.1% that are not mentioned in the product information are not shown in the table.

| **Ozanimod** (frequency according to product information) | **Adverse events** | **n** | **%** |
| --- | --- | --- | --- |
| Very common (>1/10) | Nasopharyngitis | 1 | 0.28 |
|  | Lymphopenia | 6 | 1.69 |
| Common  (≥1/100 to <1/10) | Herpes simplex | 1 | 0.28 |
|  | Orthostatic hypotension | 1 | 0.28 |
|  | Edema | 1 | 0.28 |
|  | Pulmonary function test abnormal | 1 | 0.28 |
|  | Elevated hepatic enzymes | 3 | 0.84 |
|  | Hypertension | 6 | 1.69 |
| Uncommon  (≥1/1,000 to <1/100) | Hypersensitivity | 2 | 0.56 |
| Not mentioned in the product information | cervical intraepithelial neoplasia (CIN II) | 1 | 0.28 |
|  | Basal cell carcinoma | 1 | 0.28 |
|  | Melanoma | 1 | 0.28 |
|  | Insomnia | 1 | 0.28 |
|  | Weight gain | 1 | 0.28 |
|  | Diarrhea | 1 | 0.28 |
|  | Depression | 2 | 0.56 |
|  | Alopecia | 2 | 0.56 |
|  | Leukopenia | 2 | 0.56 |

Supplementary Table 4: Frequency of AE with ozanimod in the AMSTR cohort compared with the frequency stated in the product information.

| **Ponesimod** (frequency according to product information) | **Adverse events** | **n** | **%** |
| --- | --- | --- | --- |
| Common  (≥1/100 to <1/10) | Urinary tract infection | 1 | 0.54 |
|  | Fatigue | 1 | 0.54 |
|  | Edema | 1 | 0.54 |
|  | Dizziness | 1 | 0.54 |
|  | Dyspnea | 2 | 1.08 |
|  | Lymphopenia | 3 | 1.63 |
|  | Elevated hepatic enzymes | 3 | 1.63 |
| Not mentioned in the product information | Herpes labialis | 1 | 0.54 |
|  | Palpitations | 1 | 0.54 |
|  | Nausea | 2 | 1.08 |
|  | Headache | 3 | 1.63 |

Supplementary Table 5: Frequency of AE with ponesimod in the AMSTR cohort compared with the frequency stated in the product information.

| **Siponimod** (frequency according to product information) | **Adverse events** | **n** | **%** |
| --- | --- | --- | --- |
| Very common (>1/10) | Headache | 3 | 1.5 |
|  | Elevated hepatic enzymes | 4 | 2.0 |
| Common  (≥1/100 to <1/10) | Basal cell carcinoma | 1 | 0.5 |
|  | Nausea | 2 | 1.0 |
|  | Dizziness | 2 | 1.0 |
|  | Edema | 2 | 1.0 |
|  | Lymphopenia | 9 | 4.5 |
| Uncommon  (≥1/1,000 to <1/100) | Melanoma | 1 | 0.5 |
|  | Squamous cell carcinoma | 1 | 0.5 |
| Not mentioned in the product information | Herpes genitalis | 1 | 0.5 |
|  | Leukopenia | 1 | 0.5 |
|  | Sinusbradycardia | 1 | 0.5 |
|  | Palpitations | 1 | 0.5 |
|  | Fatigue | 1 | 0.5 |
|  | retinal hemorrhage | 1 | 0.5 |
|  | Respiratory infection | 5 | 2.5 |

Supplementary Table 6: Frequency of AE with siponimod in the AMSTR cohort compared with the frequency stated in the product information.

| **Natalizumab** (frequency according to product information) | **Adverse events** | **n** | **%** |
| --- | --- | --- | --- |
| Very common (>1/10) | Arthralgia | 11 | 0.54 |
|  | Nausea | 11 | 0.54 |
|  | Dizziness | 13 | 0.64 |
|  | Urinary tract infection | 15 | 0.74 |
|  | Infusion-related reaction (IRR) | 20 | 0.98 |
|  | Fatigue | 34 | 1.68 |
|  | Headache | 40 | 1.97 |
|  | Nasopharyngitis | 44 | 2.17 |
| Common  (≥1/100 to <1/10) | Hepatic enzyme increased | 4 | 0.19 |
|  | Pruritus | 5 | 0.24 |
|  | Anaemia | 7 | 0.34 |
|  | Rash | 9 | 0.44 |
|  | Allergic reaction | 25 | 1.23 |
|  | Herpes infection | 28 | 1.38 |
| Uncommon  (≥1/1,000 to <1/100) | Anaphylactic reaction | 4 | 0.19 |
|  | Progressive multifocal leukoencephalopathy | 7 | 0.34 |
| rare (≥1/10,000 to <1/1,000) | Nucleated red blood cells | 1 | 0.06 |
| Not mentioned in the product information | Inflammation | 3 | 0.14 |
|  | Thrombosis | 3 | 0.14 |
|  | Edema | 3 | 0.14 |
|  | Seizure | 3 | 0.14 |
|  | Mood swings | 3 | 0.14 |
|  | Weight loss | 3 | 0.14 |
|  | Concentration difficulty | 4 | 0.19 |
|  | Eczema | 4 | 0.19 |
|  | Pneumonia | 5 | 0.24 |
|  | Diarrhea | 5 | 0.24 |
|  | Influenza-like symptoms | 5 | 0.24 |
|  | Alopecia | 7 | 0.34 |
|  | Lymphocytosis | 8 | 0.39 |
|  | Leukocytosis | 9 | 0.44 |

Supplementary Table 7: Frequency of AE with natalizumab in the AMSTR cohort compared with the frequency stated in the product information. AE with a relative frequency <0.1% that are not mentioned in the product information are not shown in the table.

| **Ocrelizumab** (frequency according to product information) | **Adverse events** | **n** | **%** |
| --- | --- | --- | --- |
| Very common (>1/10) | Infusion related reaction (IRR) | 4 | 0.57 |
|  | Upper respiratory tract infection | 9 | 1.28 |
| Common  (≥1/100 to <1/10) | Herpes infection | 3 | 0.42 |
|  | Neutropenia | 4 | 0.57 |
| Not mentioned in the product information | Nausea | 1 | 0.14 |
|  | Dyspnoea | 1 | 0.14 |
|  | Heat sensation | 1 | 0.38 |
|  | Glioblastoma | 1 | 0.38 |
|  | Acne | 1 | 0.14 |
|  | Influenza-like symptoms | 1 | 0.14 |
|  | IgM Lambda band | 1 | 0.14 |
|  | Immune thrombocytopenia | 1 | 0.14 |
|  | Esophageal candidiasis | 1 | 0.14 |
|  | Concentration difficulty | 1 | 0.14 |
|  | Fatigue | 1 | 0.14 |
|  | Rectal carcinoma | 1 | 0.14 |
|  | Edema | 1 | 0.14 |
|  | Lymphopenia | 2 | 0.28 |
|  | Hyperthyroidism | 2 | 0.28 |
|  | Folliculitis | 2 | 0.28 |
|  | Allergic reaction | 2 | 0.28 |
|  | Hepatopathy | 2 | 0.28 |
|  | Hepatic enzyme increased | 2 | 0.28 |
|  | Dizziness | 3 | 0.42 |
|  | Urinary tract infection | 3 | 0.42 |
|  | Headache | 4 | 0.57 |
|  | Leukopenia | 5 | 0.71 |
|  | Flush | 5 | 0.71 |
|  | Pruritus | 6 | 0.57 |
|  | Rash | 10 | 1.43 |

Supplementary Table 8: Frequency of AE with ocrelizumab in the AMSTR cohort compared with the frequency stated in the product information.

| **Ofatumumab** (frequency according to product information) | **Adverse events** | **n** | **%** |
| --- | --- | --- | --- |
| Very common (>1/10) | Urinary tract infection | 2 | 0.25 |
|  | Upper respiratory tract infection | 3 | 0.37 |
|  | Injection-site reactions (systemic) | 6 | 0.75 |
| Common  (≥1/100 to <1/10) | Blood immunoglobulin M decreased | 2 | 0.25 |
|  | Oral herpes | 5 | 0.63 |
| Not mentioned in the product information | Prostatitis | 1 | 0.12 |
|  | Mammary duct papilloma | 1 | 0.12 |
|  | Pituitary adenoma | 1 | 0.12 |
|  | Raynoud-syndrome | 1 | 0.12 |
|  | Exanthem | 1 | 0.12 |
|  | Tinea pedis | 1 | 0.12 |
|  | Onychomycosis | 1 | 0.12 |
|  | Sicca symptoms (eye) | 1 | 0.12 |
|  | Diarrhea | 1 | 0.12 |
|  | Elevated hepatic enzymes | 1 | 0.12 |
|  | Lymphopenia | 1 | 0.12 |
|  | Leucopenia | 1 | 0.12 |
|  | Fatigue | 1 | 0.12 |
|  | Headache | 1 | 0.12 |
|  | Other herpes | 2 | 0.25 |
|  | Pneumonia | 2 | 0.25 |
|  | Influenza-like symptoms | 3 | 0.37 |

Supplementary Table 9: Frequency of AE with ofatumumab in the AMSTR cohort compared with the frequency stated in the product information.

| **Teriflunomide** (frequency according to product information) | **Adverse events** | **n** | **%** |
| --- | --- | --- | --- |
| Very common (>1/10) | Nausea | 3 | 0.38 |
|  | Headache | 9 | 1.15 |
|  | Diarrhoea | 38 | 4.86 |
|  | Alopecia | 60 | 7.68 |
| Common  (≥1/100 to <1/10) | Mild infection | 1 | 0.12 |
|  | Menorrhagia | 1 | 0.12 |
|  | Acne | 1 | 0.12 |
|  | Paraesthesia | 1 | 0.12 |
|  | Anxiety | 1 | 0.12 |
|  | Mild allergic reaction | 2 | 0.25 |
|  | Pancreatitis | 2 | 0.25 |
|  | Myalgia | 2 | 0.25 |
|  | Arthralgia | 2 | 0.25 |
|  | Palpitations | 2 | 0.25 |
|  | Weight decrease | 3 | 0.38 |
|  | Abdominal pain | 3 | 0.38 |
|  | Urinary tract infection | 3 | 0.38 |
|  | Gastrointestinal infection | 4 | 0.51 |
|  | Leukopenia | 5 | 0.64 |
|  | Exanthem | 5 | 0.64 |
|  | Sinusitis/Pharyngitis/Laryngitis | 5 | 0.64 |
|  | Herpes infection | 8 | 1.02 |
|  | Influenza/Rhinitis/Bronchitis | 9 | 1.15 |
|  | GGT/ AST increase | 16 | 2.04 |
|  | Hypertension | 36 | 4.60 |
| Uncommon  (≥1/1,000 to <1/100) | Thrombocytopenia | 1 | 0.12 |
|  | Nail disorders | 2 | 0.25 |
|  | Aphthae | 2 | 0.25 |
|  | Colitis | 3 | 0.38 |
|  | Peripheral neuropathy | 6 | 0.76 |
| Not mentioned in the product information | Concentration difficulty | 1 | 0.12 |
|  | Insomnia | 1 | 0.12 |
|  | General malaise | 1 | 0.12 |
|  | Dyspnea | 1 | 0.12 |
|  | Cough | 1 | 0.12 |
|  | Liver failure | 1 | 0.12 |
|  | Obstructive bronchitis | 1 | 0.12 |
|  | Chest discomfort | 1 | 0.12 |
|  | Double vision | 1 | 0.12 |
|  | Blurred vision | 1 | 0.12 |
|  | Other visual disturbance | 1 | 0.12 |
|  | Hypothyroidism | 1 | 0.12 |
|  | Sexual dysfunction | 1 | 0.12 |
|  | Reflux | 1 | 0.12 |
|  | Epididymitis | 1 | 0.12 |
|  | Viral menigitis | 1 | 0.12 |
|  | Other GI-infection | 1 | 0.12 |
|  | Gonarthritis | 1 | 0.12 |
|  | Mycosis | 1 | 0.12 |
|  | Dysplastic nevus | 1 | 0.12 |
|  | Polyp | 1 | 0.12 |
|  | Fatigue | 1 | 0.12 |
|  | Spondylodiscitis | 1 | 0.12 |
|  | Depression | 1 | 0.12 |
|  | Fecal incontinence | 1 | 0.12 |
|  | Fatigue | 2 | 0.25 |
|  | Eczema | 2 | 0.25 |
|  | Esophagitis | 2 | 0.25 |
|  | Urinary incontinence | 2 | 0.25 |
|  | Lymphopenia | 3 | 0.38 |
|  | Meteorism | 3 | 0.38 |
|  | Loose stool | 3 | 0.38 |
|  | Vertigo | 5 | 0.64 |

Supplementary Table 10: Frequency of AE with teriflunomide in the AMSTR cohort compared with the frequency stated in the product information.
